# Reconstruction of the lipid metabolism for the microalga *Monoraphidium neglectum* from its genome sequence reveals characteristics suitable for biofuel production

**DOI:** 10.1186/1471-2164-14-926

**Published:** 2013-12-28

**Authors:** Christian Bogen, Arwa Al-Dilaimi, Andreas Albersmeier, Julian Wichmann, Michael Grundmann, Oliver Rupp, Kyle J Lauersen, Olga Blifernez-Klassen, Jörn Kalinowski, Alexander Goesmann, Jan H Mussgnug, Olaf Kruse

**Affiliations:** 1 Department of Biology/Center for Biotechnology, Bielefeld University, Universitätsstrasse 27, Bielefeld 33615, Germany

**Keywords:** *M. neglectum* genome, Biofuels, Lipid metabolism, Neutral lipid accumulation

## Abstract

**Background:**

Microalgae are gaining importance as sustainable production hosts in the fields of biotechnology and bioenergy. A robust biomass accumulating strain of the genus *Monoraphidium* (SAG 48.87) was investigated in this work as a potential feedstock for biofuel production. The genome was sequenced, annotated, and key enzymes for triacylglycerol formation were elucidated.

**Results:**

*Monoraphidium neglectum* was identified as an oleaginous species with favourable growth characteristics as well as a high potential for crude oil production, based on neutral lipid contents of approximately 21% (dry weight) under nitrogen starvation, composed of predominantly C18:1 and C16:0 fatty acids. Further characterization revealed growth in a relatively wide pH range and salt concentrations of up to 1.0% NaCl, in which the cells exhibited larger structures. This first full genome sequencing of a member of the Selenastraceae revealed a diploid, approximately 68 Mbp genome with a G + C content of 64.7%. The circular chloroplast genome was assembled to a 135,362 bp single contig, containing 67 protein-coding genes. The assembly of the mitochondrial genome resulted in two contigs with an approximate total size of 94 kb, the largest known mitochondrial genome within algae. 16,761 protein-coding genes were assigned to the nuclear genome. Comparison of gene sets with respect to functional categories revealed a higher gene number assigned to the category “carbohydrate metabolic process” and in “fatty acid biosynthetic process” in *M. neglectum* when compared to *Chlamydomonas reinhardtii* and *Nannochloropsis gaditana*, indicating a higher metabolic diversity for applications in carbohydrate conversions of biotechnological relevance.

**Conclusions:**

The genome of *M. neglectum,* as well as the metabolic reconstruction of crucial lipid pathways, provides new insights into the diversity of the lipid metabolism in microalgae. The results of this work provide a platform to encourage the development of this strain for biotechnological applications and production concepts.

## Background

Phototrophic microalgae are increasingly investigated for their use in biotechnological applications as these unicellular organisms offer the opportunity of introducing sustainable production pathways by converting sunlight energy and CO_2_ into valuable products [1-5]. In order to establish highly efficient strains, however, the systematic genome analysis and the development of molecular tools for genetic engineering approaches are necessary. Consequently the genomes of a number of strains of interest have recently been sequenced and essential genetic tools have been successfully introduced [6-8].

A particular focus has been placed on species with high oil content. Many microalgae are reported to produce considerable amounts of oils [2,3], where the compound of interest for biofuels is mainly triacylglycerol (TAG) [2]. TAG consists of three fatty acids esterified to a glycerol backbone [3,5]. The accumulation of TAG in microalgae usually occurs under environmental stress conditions such as high-light or nitrogen starvation [2,9] but, however, can be also increased artificially, for example, by inhibition of starch synthesis [10]. Though the lipid metabolism in algae and plants is considered to be comparatively similar, a number of differences can be found. For example, in contrast to unicellular microalgae, TAG synthesis in plants predominantly takes place in specialized tissues or organs [2]. Furthermore, current results indicate not only the presence of clear differences between plants and microalgae, but also between different microalgal species [11]. Microalgae exhibit high levels of diversity between species due to their different evolutionary history, thus it is expected that the lipid metabolism amongst the various strains also exhibits distinct differences. This is exemplified by the comparison of the green algal model organism *Chlamydomonas reinhardtii* with *Nannochloropsis *[11]. Variable lipid metabolism within the microalgae is also suggested by the high diversity of lipids of different classes and unusual fatty acids found in individual algae strains, even among the same division [12,13].

Based on this knowledge, numerous systematic screens aiming to identify microalgal species with high lipid content have been performed during recent years [14-19]. With the identification of strains of interest by these approaches, the necessity for systematic analyses of genomes by next-generation sequencing, annotation and reconstruction of lipid metabolic pathways becomes evident. These strategies allow deeper insights into the lipid metabolism and evolutionary strategies of these photosynthetic microorganisms.

The genome of the green algal model organism *C. reinhardtii* has been sequenced [8], as well as the genome of the TAG-accumulating Eustigmatophytes *Nannochloropsis gaditana *[20,21], *Nannochloropsis oceanica *[6], and several other microalgae [22].

In our laboratory, we recently succeeded in identifying a strain of the genus *Monoraphidium* with high fatty acid abundances combined with robust biomass accumulation through a screening strategy which combined up-scaling tests, determination of total lipids, and the evaluation of fatty acid abundances [19]. The identification of *Monoraphidium contortum* from this screening is in coincidence with rising interest in this genus over recent years [23,24]. Strains of the genus *Monoraphidium* belong to the family of the *Selenastraceae* within the class *Chlorophyceae*. So far, little information is available on genomes of members of the family *Selenastraceae* and related species such as *Scenedesmus obliquus*. In the light of the fact that the biodiversity of microalgae is tremendous, much of the potential for strain identification and characterisation to contribute to liquid biofuel purposes remains to be explored [2].

Based on 18S rRNA analysis, it was shown that species of this genus tightly cluster with species of *Ankistrodesmus*, *Kirchneriella*, and other genera, thus not forming a monophyletic group [25]. When *M. contortum,* isolated from a natural habitat in Thailand, was grown in different autotrophic media, it showed comparatively high lipid productivities among the strains under the investigated conditions [23].

*Monoraphidium neglectum* was also identified as a robust strain belonging to the family Selenastraceae, by our previous screening efforts [19]. *M. neglectum* was shown to cluster differently to *M. contortum *[24], on the same branch as *Podohedriella falcata *[25], also known as *Ankistrodesmus falcatus*. Another strain of this species was reported as an oleaginous organism before [26], highlighting the need for more detailed comparisons and investigations among the members of the *Selenastraceae* by metabolomic studies and whole genome sequencing. When directly compared to *M. contortum*, *M. neglectum* showed some phenotypical characteristics such as higher robustness in up-scale cultivations, making this strain even more interesting for biotechnological applications.

In this work, we intensively characterised the oleaginous phenotype of *Monoraphidium neglectum* and established and annotated a draft genome of this organism as a precondition for metabolic network reconstruction. Investigation of the reconstructed metabolic pathways with respect to key enzymes for triacylglycerol formation was carried out, setting the basis for further investigations and offering the possibility to develop strategies for genetic improvements. In addition, *M. neglectum* was further biochemically characterised as a robust production strain suitable for biotechnological approaches.

## Results

The species *M. neglectum* was identified in a previous screen for oleaginous microalgal strains that exhibited robust growth characteristics at varying cultivation conditions [19]. To evaluate the biotechnological potential of this strain, a detailed investigation was performed in this work. This investigation included detailed physiological analysis, evaluating lipid yields and robustness. Genome sequencing and annotation were performed and combined with the reconstruction of phylogeny as well as an analysis of lipid metabolism pathways, features crucial for understanding and establishing *M. neglectum* as lipid-producing feedstock.

### *M. neglectum* exhibits a rapid photoautotrophic growth phenotype and accumulates neutral lipids to a high extent under nitrogen starvation

**Figure 1:**
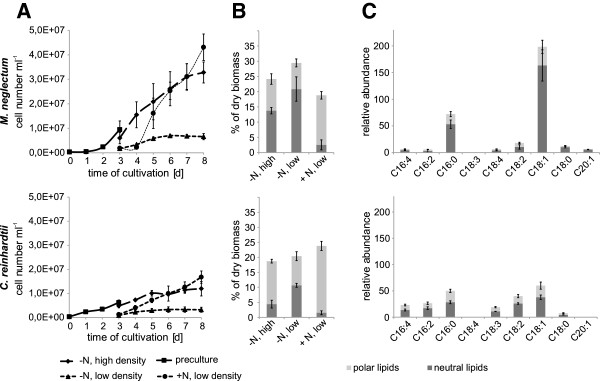
**Comparison of growth and lipid accumulation of *Monoraphidium neglectum *with *Chlamydomonas reinhardtii. *(A)** Growth under nitrogen replete (+N) and nitrogen deficient (-N) conditions at two different culture densities (high and low density) (n = 6). (**B)** Neutral and polar lipid content as determined after modified Folch extraction and subsequent chromatography after five days of cultivation (n = 4). **(C)** Relative abundances of fatty acids (determined by comparison to internal C17-FA standard set to a value of 100) of *M. neglectum* (top) and *C. reinhardtii* (bottom) after nitrogen starvation for five days in low culture densities (-N, low density), (n = 4). Error bars indicate standard deviation.

The combination of efficient phototrophic biomass accumulation and high neutral lipid content is considered as one of the most essential traits of an algal strain used for liquid biofuel production [17]. Besides nutrient starvation, light stress is an important trigger in the generation of triacylglycerols [2]. Therefore, both factors were investigated in a combined approach. As in previous work, the model alga *C. reinhardtii* was used as a control. Cells of both species were inoculated to the same biomass density and cultivated phototrophically for three days to obtain comparable starter cultures (Figure 1, precultures). After three days, cells were harvested and used for comparative analyses. To assess lipid accumulation induced by nutrient starvation, cells were resuspended in nitrogen replete medium or in medium deficient in nitrogen (Figure 1, +N or –N, respectively). To assess additional effects related to light availability, two cell densities were chosen for the –N samples and designated “low density” or “high density”, respectively.

For *M. neglectum*, low density cultures corresponded to 0.22 g l^-1^ (2 × 10^6^ ± 5 × 10^5^ cells ml^-1^) and high density cultures corresponded to 0.78 g l^-1^ (6 × 10^6^ ± 2 × 10^6^ cells ml^-1^), For the reference strain *Chlamydomonas reinhardtii,* similar cell densities were chosen. Biomass accumulation was subsequently monitored and lipid contents as well as fatty acid compositions were determined after five days of cultivation (Figure 1).

The neutral lipid content (per dry weight) increased from initial 1.4 ± 0.6% (+N, preculture) to up to 20.9 ± 4.0% upon nitrogen starvation in low-density cultures in *M. neglectum* (Figure 1B).

The overall neutral lipid productivity was determined during the two-step cultivation process for the high density cultures. In this condition, *M. neglectum* reached maximal productivity rates of 52 ± 6 mg neutral lipids l^-1^ day^-1^, which was four times higher compared to *C. reinhardtii* (13 ± 4 mg neutral lipids l^-1^ day^-1^, see Additional file 1: Figure S1), a finding that could, in particular, be attributed to a pronounced increase of biomass in the beginning of the starvation period in the case of *M. neglectum*. The relative abundance of fatty acids was determined (with an internal C17-FA standard set to a value of 100) to directly compare the individual fatty acid accumulation between *M. neglectum* and *C. reinhardtii. M. neglectum* reached comparatively high overall abundances of fatty acids in the total lipid fraction under nitrogen starvation that were roughly 50% higher than those detected in *C. reinhardtii* (Figure 1C, Additional file 2: Figure S2). While the total abundance of fatty acid increased only 1.1-fold in high density and 1.5-fold in low density cultures in *C. reinhardtii* when compared to the nutrient replete control (Figure 1C, Additional file 2: Figure S2), *M. neglectum* showed a 2-fold and 2.5-fold increase of its fatty acid content in high and low density cultures respectively (Figure 1C, Additional file 2: Figure S2). When only the fatty acids of the neutral lipid fraction were considered, this finding became even more evident.

In *M. neglectum*, the fatty acid abundances encountered in the neutral lipid fraction of N-starved cells of low density cultures were about tenfold higher in comparison to those of the non-starved cultures (Figure 1C, Additional file 2: Figure S2). This accumulation pattern is similar to the observation in *C. reinhardtii*, where fatty acids of the neutral lipid fraction increased more than tenfold (Figure 1C, Additional file 2: Figure S2) in cultures with lower biomass densities when compared to the nutrient replete control. *M. neglectum*, therefore, accumulated neutral lipids and fatty acids to high abundances under nitrogen limitation, a feature which was enhanced with increased light penetration. These cultivation parameters also resulted in a positive effect on the accumulation of fatty acids within the neutral lipid fraction in the investigated strains. Interestingly, both strains exhibited a decrease of fatty acids in the polar lipid fraction when nitrogen starvation at low culture densities was applied (Figure 1C, Additional file 2: Figure S2), an observation also reported for other strains [2].

In *M. neglectum*, the increase of the overall fatty acid content under nitrogen starvation could be mostly attributed to an accumulation of C18:1 and C16:0 which contributed considerably to the neutral lipid fraction (Figure 1C). This finding is also reflected by the gravimetrical determinations which show a pronounced increase of the neutral lipid fraction, while the total lipid content increased only moderately (Figure 1B).

In contrast, *C. reinhardtii* demonstrated a more diverse accumulation pattern of fatty acids in the neutral lipid fraction under nitrogen starvation, where not only saturated C16 and monoenoic C18 but also C16:4, C18:3 and dienoic C18 abundances increased (Figure 1C).

It is well established that microalgal biomass and lipid production are strongly dependent on the cultivation conditions [27]. It was, therefore, important to compare the production rates of *M. neglectum* with other known oleaginous species in the chosen setup. *Parachlorella kessleri* and *Scenedesmus obliquus* were selected for comparative analyses because they represent prominent microalgal species with superior lipid productivities [17,28]. Low density conditions were applied for all strains to achieve maximum lipid accumulation levels (Table 1).

**Table 1:** **Biomass productivities and lipid content of *Monoraphidium neglectum *compared to other microalgal species**

	**Biomass production (max) [g**^**-1**^ **l**^**-1 **^**day]**	**Biomass production (mean) [g l**^**-1 **^**day**^**-1**^**]**	**Lipid contents (non-starved) [% DW] Total lipids/Neutral lipids**	**Lipid content (N-Starvation) [% DW] Total lipids/Neutral lipids**
*Monoraphidium neglectum*	0.967	0.603	21 ± 1.7 / 2.5 ± 1.6	30 ± 3.4 / 20.9 ± 4.0
*Chlamydomonas reinhardtii*	0.575	0.363	24 ± 1.4 / 1.5 ± 0.7	20 ± 0.8 / 10.7 ± 0.7
*Parachlorella kessleri*	0.650	0.435	22.6 / 1.2	33.9 / 18.0
*Scenedesmus obliquus*	0.725	0.435	17.4 / 1.4	37.9 / 21.5

Biomass productivities and lipid contents from nitrogen replete and N-starved low density cultures. Standard deviation is shown from 4 replicates for *M. neglectum* and *C. reinhardtii*, and means of two replicates for *P. kessleri* and *S. obliquus*.

When directly compared, *M. neglectum* demonstrated the highest overall biomass productivity, exceeding those of all three control strains by 33 - 68% under optimal nutrient replete conditions (Table 1).

After nitrogen starvation, *M. neglectum* had a maximal total lipid content of up to 30 ± 3.4% of dried biomass, significantly higher than in the control strain *C. reinhardtii* with only 20 ± 0.8% (Table 1). The total lipid amount in the oleaginous species *P. kessleri* and *S. obliquus* was determined to be higher in this setup; however, the neutral lipid content of *M. neglectum* was comparable to the top performer *S. obliquus.* The results demonstrate that neutral lipid production of *M. neglectum* can be at least equal to *P. kessleri* and *S. obliquus*. Therefore, *M. neglectum* represents a new oleaginous microalgal species.

### *M. neglectum* shows a robust growth phenotype with a high salt and pH tolerance during phototrophic growth

**Figure 2:**
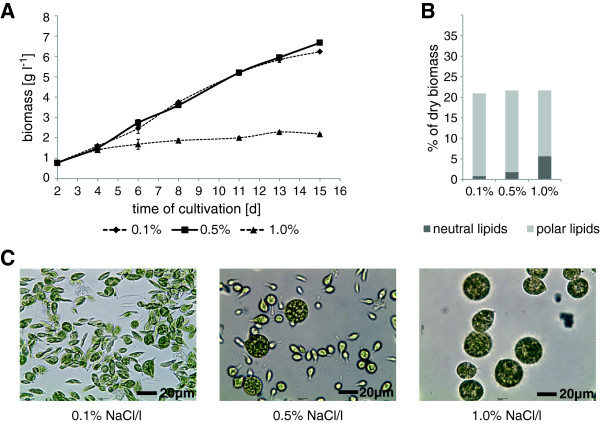
**Salt tolerance evaluation of *M. neglectum.*** Cultivations were performed with 0.1% (salt concentration of the standard medium), 0.5% or 1% (w/v) sodium chloride. **(A)** Biomass accumulation at 350 - 400 μmol photons m^-2^ s^-1^ constant light, error bars indicate standard deviation (n = 3). **(B)** Neutral and polar lipid content in percent of dry biomass of two replicates. **(C)** Light microscopic images, black bar corresponds to 20 μm.

The ability to support growth in brackish or marine environments is an important trait for strains considered for large scale outdoor cultivations. Salt tolerance and cellular adaptation reactions were systematically evaluated in terms of growth, lipid content and composition as well as cell morphology. As shown in Figure 2A, salt concentrations of 0.5% (w/v) did not affect the biomass accumulation negatively. When salt concentrations were increased to 1%, cells still survived but biomass accumulation was severely reduced.

Neutral lipid contents were found to increase with the induction of salt stress, reaching a maximal content of 8.3% (dry weight) for the cultures grown at 1% salt concentration (Figure 2B). Furthermore, a clear effect of the salt concentration on the cell shape and size was found (Figure 2C). While cells exhibited a mace-like shape with an average size of about 10 μm at lower salt concentrations, larger round cell clusters of about 25 μm were formed and sustained when the salt content of the media increased, potentially mitigating the salt stress by decreasing the cellular surface to volume ratio.

**Figure 3:**
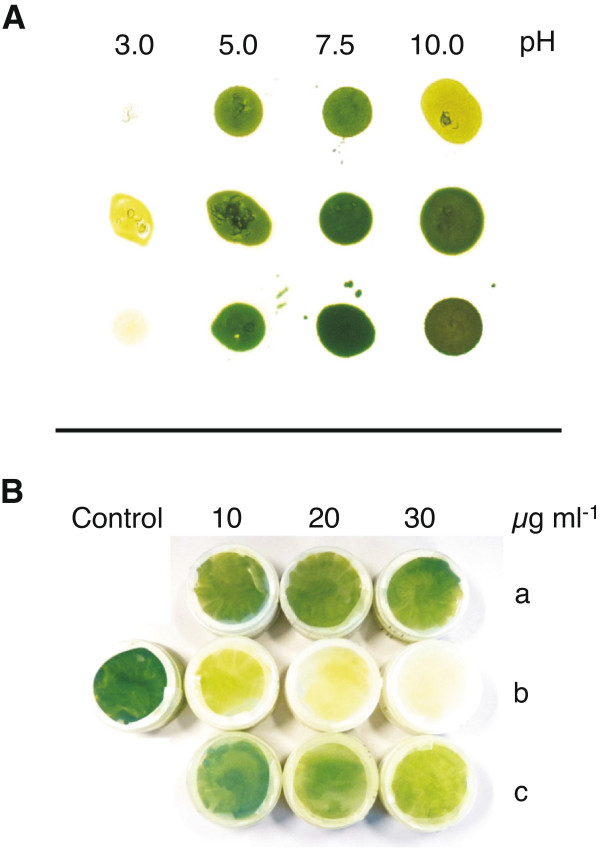
**Investigation of growth on *M. neglectum *at different pH and with different antibiotics when grown on agar plates under constant illumination. (A)** Test of pH tolerance for *M. neglectum*. Rows indicate the three biological replicates. **(B)** Culture sensitivity to antibiotics in ProF media supplemented with 1% glucose. Three different concentrations of (a) kanamycin, (b) hygromycin and (c) paromomycin, control without antibiotics.

It is well known that the pH can be affected during prolonged microalgal cell cultivation, for example, by secreted fermentation products. For a production strain, tolerance to pH differences can, therefore, be advantageous. In addition, cultivation at higher pH increases the efficiency of CO_2_ fixation and may decrease bacterial contamination under non-sterile cultivation conditions. Plate assays were performed to evaluate the sensitivity of *M. neglectum* to a varying pH range. *M. neglectum* exhibited tolerance to a wide range of pH conditions and was able to grow between pH 5 and pH 10 (Figure 3A).

The fact that *M. neglectum* was able to tolerate salt concentrations found in brackish water and was furthermore able to cope with a range of pH conditions demonstrated its generally robust growth characteristics.

As antibiotics are frequently used in the generation of axenic cultures, as well as selective agents for gene transformation, we investigated whether *M. neglectum* could tolerate antibiotics commonly used for these strategies (Figure 3B). Concentrations of kanamycin, hygromycin B, and paromomycin ranging from 10-30 μg ml^-1^ were applied to plate-level cultures under mixotrophic growth. Although all three substances belong to the group of aminoglycoside antibiotics, significant differences regarding cell toxicity were observed. *M. neglectum* demonstrated antibiotic resistance against kanamycin with concentrations up to 30 μg ml^-1^, whereas severe sensitivities to hygromycin B were observed already at 10 μg ml^-1^. The strain also demonstrated resistance to paromomycin. Consequently, powerful antibiotics are available as tools to decontaminate *M. neglectum* cultures (kanamycin, paromomycin) or as selection reagents for future transformation approaches (hygromycin B).

### Full genome sequencing and assembly reveals the diploid character of the *M. neglectum* genome

The genome of *M. neglectum* was sequenced by next-generation sequencing techniques to elucidate its metabolic pathways and to lay the foundation for a detailed genetic analysis. The genome sequence was obtained using the Illumina MiSeq^©^ technology yielding paired-end reads of 2 × 250 bases in length. Over 8 million paired reads were assembled, resulting in about 6,700 scaffolds: thereof 857 were longer than 20 kb (Table 2).

**Table 2:** **Assembly statistics for nuclear draft genome of *M. neglectum***

Estimated genome size	~ 68 Mb
Genomic G + C content	64.74%
Coverage	49.30
Aligned reads	16,194,053
Number of assembled scaffolds	6,739
Number of scaffolds > 20 kb in length	857
Scaffold N50	1,303
Scaffold L50	15,659
Number of contigs	25,211
Number of contigs > 20 kb in length	11
Number of contigs > 2 kb in length	2103
Scaffolded contig N50	2,150
Scaffolded contig L50	9,165

**Figure 4:**
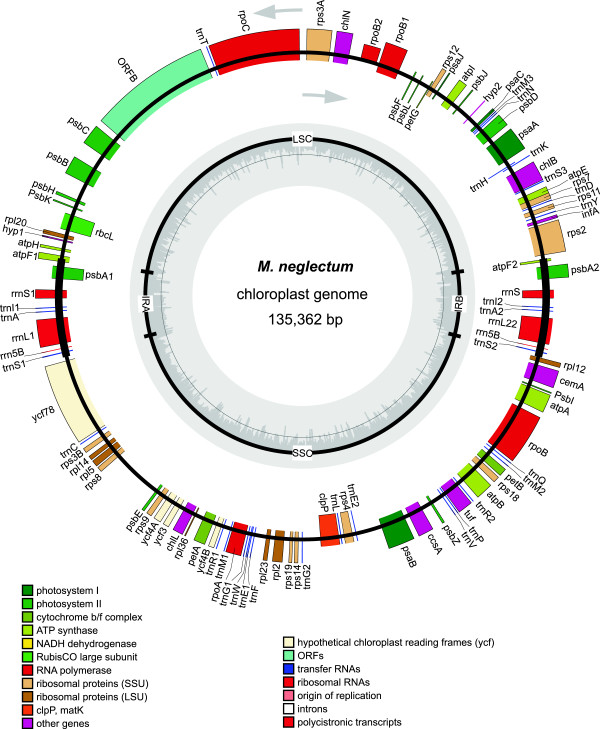
**Chloroplast genome of *Monoraphidium neglectum*.** Visualization of the DNA sequence was performed via OrganellarGenomeDRAW (OGDRAW) [30].

**Figure 5:**
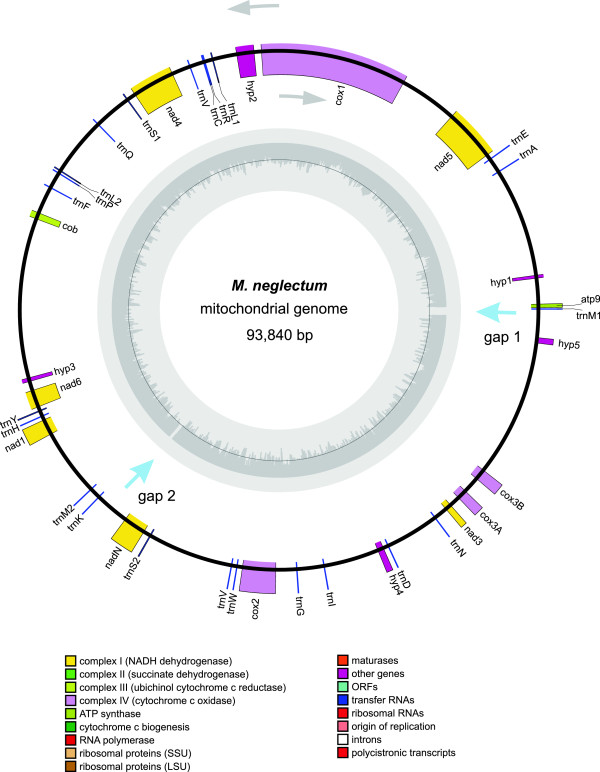
**Mitochondrial genome of *Monoraphidium neglectum*.** Visualization of the mitochondrial genome via OrganellarGenomeDRAW (OGDRAW) [30].

The investigation of contig-length vs. read-count (Additional file 3: Figure S3) as performed according to [29] revealed a diploid character of the sequenced genome showing homozygous and heterozygous contigs. To identify the contigs and scaffolds constituting the chloroplast and mitochondrion of *M. neglectum*, a tBLASTx search was carried out using genes of other known chloroplast and mitochondrial genomes. The chloroplast genome was assembled into a single circular contig with a size of 135,362 bp (Figure 4) and a G+C content of 32.38%. The chloroplast genome contains the large single copy (LSC), the small single copy (SSC), as well as inverted repeat (IR) regions (Figure 4). The assembly of the mitochondrial genome led to two contigs separated by gaps (Figure 5). Since the gaps are in-between scaffolded contigs, it is certain that the mitochondrial genome is circular. The mitochondrial genome has a size of approximately 94 kb and a G+C content of 45.32%. The remaining scaffolds add to an estimated nuclear genome size of approximately 68 Mb with a G+C content of 64.7% (Table 2).

### Genome annotation identifies *M. neglectum* as member of the Selenastraceae with a large mitochondrial genome

Gene prediction was performed with different bioinformatics approaches. Prediction by Augustus [31], trained with the *C. reinhardtii* genome, resulted in 13,930 potential genes and prediction by GeneMark-ES [32] in 20,149. Both predictions were analysed by EVidenceModeler [33], integrating BLASTp protein alignment data with *C. reinhardtii*. This validation step resulted in a set of 16,845 genes (assuming one transcript per gene) (Table 3).

**Table 3:** Gene prediction statistics

Total number of genes	16,845
Coding density	25.16%
Number of nuclear genes	16,761
Chloroplast genes	67
Mitochondrial genes	17
Nuclear rRNA	ca. 115 (23 copies of a contig containing 1× 8S, 2× 28S, and 2× 18S)
Chloroplastic rRNA	6 (2× 5S, 2× 23S, and 2× 16S)
Mitochondrial rRNA	none
Nuclear tRNA	40 + 1× Pseudo Ser-tRNA
Chloroplastic tRNA	29 + 1× Pseudo Leu-tRNA
Mitochondrial tRNA	21 + 1× Pseudo Met-tRNA
Maximal coding sequence length	21,519 bp
Minimal coding sequence length	144 bp
Median coding sequence length	155 bp
Maximal intron length	7,198 bp
Minimal intron length	23 bp
Median intron length	401 bp
Number of single exon genes	3,030

During validation, 0.2% of genes predicted by Augustus and 15.4% of genes predicted by GeneMark were rejected by EVidenceModeler. Visualisation of gene predictions and further manual curation of data were performed using the in-house genome annotation system GenDB [34], modified for eukaryotic genomes (GenDBE).

To investigate the chloroplast-specific features of *M. neglectum*, the chloroplast genome was compared with those of *C. reinhardtii* and *N. gaditana* (Figure 4, Additional file 4: Figure S4). The chloroplast genome of *M. neglectum* is similar to *N. gaditana* and *C. reinhardtii* concerning the gene content. The three chloroplasts have a common set of 77 orthologous genes, including regions encoding ribosomal RNAs and tRNAs (Figure 4, Additional file 4: Figure S4). As in other chloroplast genomes, these genes encode crucial functions of photosynthesis and chloroplast-specific gene expression. Furthermore, all subunits for the synthesis of an active light-independent protochlorophyllide reductase are present.

The unique sets observed for each chloroplast genome include 12 *M. neglectum*-specific genes, 66 *N. gaditana*-specific genes, and 18 genes specific to the *C. reinhardtii* chloroplast. Several duplications of genes where found in the *M. neglectum* chloroplast, such as for the B subunit of ATP synthetase, the photosystem Q(B) protein (psbA), the photosystem I assembly protein (ycf4) and for several tRNAs. In addition, the chloroplast of *M. neglectum* exhibits specific single copy genes, such as the translation initiation factor IF-1 (encoded by infA).

Interestingly, the chloroplast genomes of the three algae have a similar gene composition, but only little synteny could be found.

This finding is supported by the large variance in the chloroplast genome size encountered among sequenced algal genomes [35,36]. In contrast, when compared to the chloroplast genomes of algae, conservation of structure and gene content of chloroplasts appears to be greater in plants.

The mitochondrial genome of *M. neglectum* is almost six times as large as the mitochondrial genome of *C. reinhardtii* (16 kb); however, it contains fewer genes and extended, potentially non-coding regions. This observation is in accordance with the understanding that algal mitochondrial genomes generally show a high degree of diversity [37]. In total, 17 genes plus 23 tRNAs were identified in the mitochondrial genome of *M. neglectum*. The basic set of mitochondrial genes is present, including subunits of the NADH dehydrogenase (nad1, nad3, nad4, nad5 and nad6), the cytochrome bc1 complex, cytochrome oxidases (cox2 and cox3) and one subunit of ATP synthase (atp9).

The 23 tRNAs observed represent an almost complete set of tRNA for translation. Only a threonine-tRNA gene is missing in the mitochondrial genome, together with ribosomal RNA genes. It is well known that the expression of mitochondrial genes is mainly dependent on the nucleus-encoded transcriptional and translational machinery [38].

The nuclear genome of *M. neglectum* contains approximately 23 copies of a contig harbouring stable RNA genes including 18S, 28S, and 8S rRNA, as well as 40 tRNAs, and one pseudo-serine tRNA. The 40 tRNAs form a complete set for protein synthesis.

The overall protein coding sequence lengths were compared between the three algal genomes. *M. neglectum* exhibits a coding sequence length of 3.156 Mb, approximately 16% greater than *C. reinhardtii* (2.7 Mb) and even more than 114% when compared to *N. gaditana* (1.473 Mb). This number is likely to be reduced by future transcriptional investigations.

To overcome fragmentations, the sequenced *Monoraphidium* genome was compared with the datasets derived from RNA sequencing approaches of the already well-investigated *C. reinhardtii* and *N. gaditana*. The comparative approach, conducted using EDGAR software [39], taking into account only genes shared between *M. neglectum* and *C. reinhardtii*, yielded 4,249 protein coding regions with an average length of 891 bp. In addition, *M. neglectum* and *N. gaditana* share 2,190 protein coding genes with an average length of 748 bp. In comparison, 5,945 proteins could be identified as singletons with an average length of only 191 bp. This finding indicates that a comparative approach can be used as a powerful tool to reduce substantially potential false positives gene predictions. These assumptions, however, require further confirmation by future RNA sequencing of *M. neglectum*.

**Figure 6:**
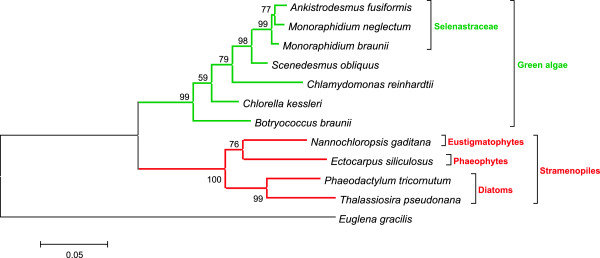
Molecular phylogenetic analysis based on the 18S rDNA sequences using the maximum likelihood method.

Phylogenetic relationship analyses confirmed that *M. neglectum* clusters within the family of Selenastraceae (Figure 6), but shows a pronounced difference in its 18S rDNA sequence when compared to *M. braunii *[25]. Selenastraceae also cluster closely to the branch of *Scenedesmus obliquus*, which was used as an oleaginous control species in this work and had been recommended for liquid biofuel applications before [17,40].

### Comparative analyses of the predicted gene functions revealed higher gene numbers assigned to carbohydrate metabolism and fatty acid biosynthesis

**Figure 7:**
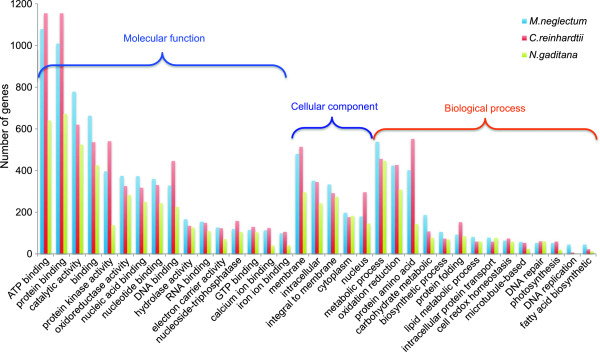
**Gene ontology (GO) assignments for *M. neglectum*, *C. reinhardtii, *and *N. gaditana*.** The 35 most extensive GO-terms of the three GO- super categories “molecular function”, “cellular component” and “biological process” are shown. Identification of gene-specific GO-terms was calculated using InterPro [41].

For a deeper investigation of gene functions of *M. neglectum* in comparison to *C. reinhardtii* and *N. gaditana,* we calculated the gene ontology terms for the complete gene-sets of the three genomes using InterPro [41]. In general, most functional categories reveal slightly lower numbers of genes in *N. gaditana*, probably reflecting the lower total gene number in this organism (Figure 7). Whereas *C. reinhardtii* shows the highest number of genes in categories such as “protein binding”, “DNA binding”, and “protein kinase activity”, suggesting a larger regulatory repertoire, *M. neglectum* exhibits a higher gene number in the category “catalytic activity”. This higher abundance of genes encoding putative metabolic functions is reflected in more detail in the categories “carbohydrate metabolic process” and “fatty acid biosynthetic process”, in which, *M. neglectum* again displays the highest gene numbers of the three genomes.

The attribution of predicted genes to gene families here provides a first, general, overview on gene families in *M. neglectum*. This, however, needs to be confirmed by more detailed functional and biochemical investigations. An important point to consider is the elimination of pseudogenes that could be identified by techniques such as transcriptome sequencing.

### Detailed genome analysis based on lipid pathway reconstruction provides essential new insights into the lipid metabolism of green microalgae

**Figure 8:**
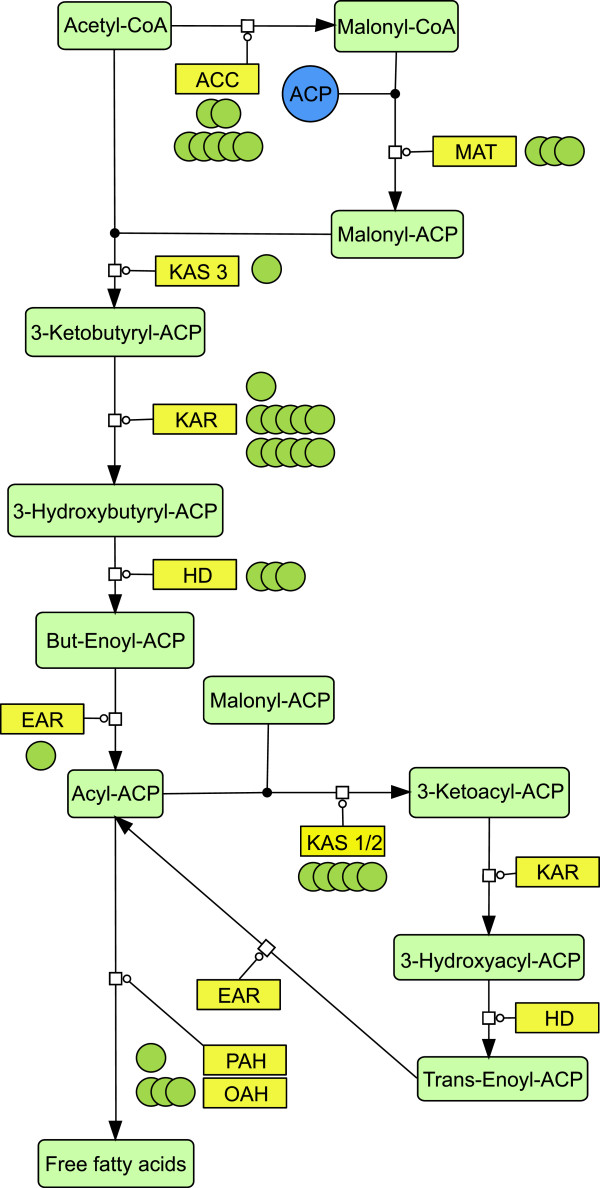
**Fatty acid synthesis pathway reconstruction based on sequence similarities.** Enzymes are indicated by yellow boxes. Each green circle indicates a homologous gene found for the respective enzyme, metabolites are indicated by light green boxes. ACC: acetyl-CoA carboxylase (EC: 6.4.1.2), MAT: acyl-carrier-protein S-malonyltransferase (EC: 2.3.1.39), KAS3: beta-ketoacyl-acyl-carrier-protein synthase III (EC: 2.3.1.180), KAR: 3-oxoacyl-acyl-carrier-protein reductase (EC: 1.1.1.100), HD: hydrolyases (EC: 4.2.1.-), EAR: enoyl-acyl-carrier-protein reductase (EC: 1.3.1.-), KAS1/2: beta-ketoacyl-acyl-carrier-protein synthase I/II (EC: 2.3.1.179, 2.3.1.41), OAH: oleoyl-acyl-carrier-protein hydrolase (EC: 3.1.2.14), PAH: palmitoyl-protein thioesterase (EC: 3.1.2.22). All identified enzymes showed an E-value <10^-10 as determined by PRIAM search described in the methods and materials section.

Metabolic pathway reconstruction was performed based on the genome data to investigate potential strategies to further improve TAG production. The pathways for fatty acid biosynthesis and the glycerolipid metabolism were reconstructed by comparison of annotated genes with KEGG database assignments. As a result, the key enzymes of the chloroplast fatty acid synthesis pathway have been identified (Figure 8).

These enzymes include the malonyl-CoA:ACP transacylase (MAT, 2.3.1.39), the beta-ketoacyl-acyl-carrier-protein synthase 3 (KAS3, EC number: 2.3.1.180), the beta-ketoacyl-acyl-carrier-protein synthase 1/2 (KAS1/2, EC number: 2.3.1.179), the 3-oxoacyl-ACP reductase (KAR, EC number: 1.1.1.100), the beta-hydroxyacyl-ACP dehydrase (HAD, EC number: 4.2.1.), and an enoyl-ACP reductase (EAR, EC 1.3.1.9).

This set of enzymes is able to catalyse the complete reaction chain from acetyl-CoA via acyl carrier proteins (ACP) to the respective fatty acyl-ACP. Notably, a comparatively high number of homologous genes were identified for acetyl-CoA carboxylase with seven genes (ACC, EC number: 6.4.1.2) and KAR with 11 genes. In addition, homologues have been identified for the fatty acyl-ACP hydrolase (OAH, EC number: 3.1.2.14, PAH, EC number: 3.1.2.22) which cleaves the fatty acid from the acyl carrier protein, releasing the free fatty acid usually as palmitate or stearate, which can then be subjected to several modifications, such as elongation or desaturation. In total, 17 fatty acid desaturase (EC 1.14.19.-) homologues were detected, including five delta-9 desaturase (EC 1.14.19.1) as well as six delta-12 desaturase (EC: 1.14.19.6) homologues.

**Figure 9:**
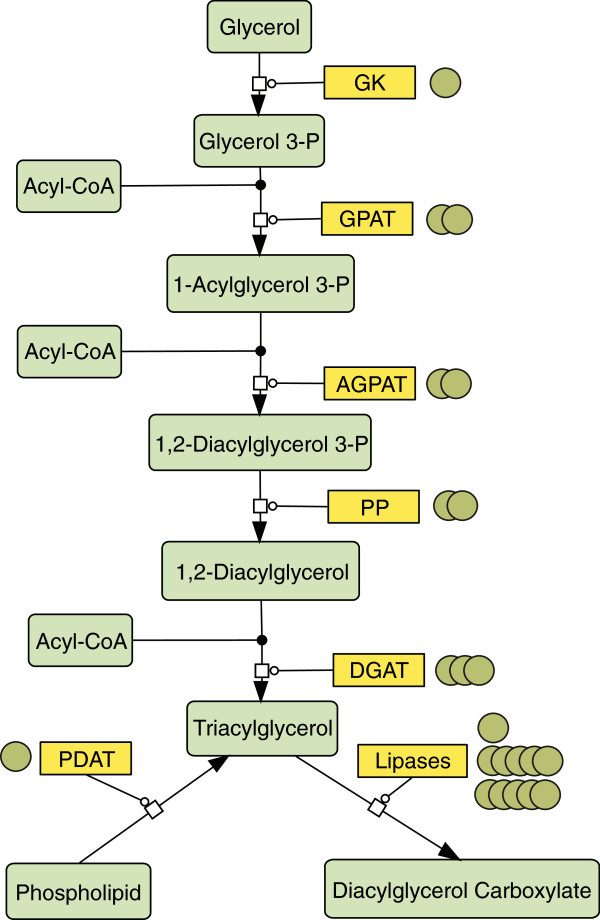
**Glycerolipid pathway reconstruction with a focus on TAG formation via the Kennedy pathway reactions.** Enzymes are indicated by yellow boxes, each green circle indicates a homologous gene found for the respective enzyme, metabolites are indicated by light green boxes. GK: glycerol kinase (EC: 2.7.1.30), GPAT: glycerol-3-phosphate O-acyltransferase (EC: 2.3.1.15), AGPAT: 1-acylglycerol-3-phosphate O-acyltransferase (EC: 2.3.1.51), PP: phosphatidate phosphatase (EC: 3.1.3.4), DGAT: acyl-CoA:diacylglycerol acyltransferase (EC: 2.3.1.20), PDAT: phospholipid:diacylglycerol acyltransferase (EC: 2.3.1.158), TAG Lipases (EC: 3.1.1.3). All identified enzymes showed an E-value <10^-10 as determined by PRIAM search described in the methods and materials section.

In addition to the fatty acid metabolism, genes coding for enzymes of the glycerolipid metabolism [42,43] were investigated in more detail. Two genes encoding proteins that show high similarity to acyl-CoA:diacylglycerol acyltransferases type 2 (DGAT2) were identified as well as one encoding a DGAT type 1 homologue. DGAT catalyses the final reaction in TAG generation in the acyl-CoA dependent pathway (Figure 9) and its orthologs have been intensely studied in several phototrophic organisms such as *Arabidopsis thaliana*, *Brassica napus,* and *C. reinhardtii *[44-47]. Other identified genes of this pathway include glycerol kinase (GK, EC 2.7.1.30), glycerol-3-phosphate O-acyltransferase (GPAT, EC 2.3.1.15), 1-acylglycerol-3-phosphate O-acyltransferase (AGPAT, EC number: 2.3.1.51), and phosphatidate phosphatase (PP, EC number: 3.1.3.4), which are responsible for the formation of lysophosphatidic and phosphatidic acid, as well as diacylglycerol, respectively (Figure 9).

The role of a further acyl-transferase, the phospholipid:diacylglycerol acyltransferase (PDAT), appears highly interesting for TAG metabolism, as reported recently [48]. PDAT mediates an acyl-CoA independent pathway to generate TAG. It was shown that PDAT of *C. reinhardtii* is able to utilise multiple substrates and performs various enzymatic reactions, ranging from TAG synthesis via transacylation of DAG with phospholipid or galactolipid acyl groups to transacylation of two DAG [48].

PDAT was shown not only to be able to hydrolyse phospholipids, galactolipids and cholesteryl esters but also TAG, therefore, attributing an important function in membrane turnover as well as TAG synthesis and degradation to this enzyme. When the genome of *M. neglectum* was analysed for PDAT homologues, two candidates were found, with one (Mneg_03131.t1) showing a conserved phosphatidylcholine-sterol O-acyltransferase domain (Figure 9), representing interesting potential targets for future biotechnological engineering approaches.

Since *M. neglectum* exhibits a strong response to nitrogen depletion, the regulation of genes involved in TAG generation as well as in the breakdown of polar lipids is of special interest. A nitrogen response regulator (NRR) has been identified in the genome of *C. reinhardtii* and was shown to be involved in neutral lipid accumulation under nitrogen depletion, as well as in the regulation of DGAT type 1 and type 2 expression [45]. A potential homologue of this regulator was found in the genome of *M. neglectum* (Mneg_07163.t1) which also exhibits a putative SQUAMOSA promoter-binding-like domain, and could, therefore, play a role in the nitrogen stress response of *M. neglectum*.

The successful reconstruction of the metabolic pathways of fatty acid and glycerolipid synthesis clearly demonstrate the integrity and quality of the genome sequencing and annotation data. These results provide new insights into the lipid metabolism of green microalgae, presenting the opportunity to identify targets for future metabolic engineering that could increase the neutral lipid production potentials of *M. neglectum*.

## Discussion

### The characterization of *M. neglectum* as a robust phototrophic oleaginous strain highlights its potential suitability for oil-based biofuel production

Stress induction by nitrogen starvation and enhanced light penetration are well-known mechanisms and driving forces for increasing neutral lipid accumulation in many microalgal strains [2,49]. *M. neglectum* exhibits a distinct accumulation of neutral lipids and the fatty acids of this fraction as response to nitrogen starvation (Table 1, Figure 1B, and C). The observation that low culture densities even increase this effect (Figure 1B), underlines the importance of light intensity for reaching optimal lipid accumulations in particular for this strain.

The importance of light for optimal lipid accumulation was already demonstrated for other algae such as *Nannochloropsis sp*. [15] and *Parachlorella kessleri *[28]. However, other investigations with *Chlorella minutissima* and *Dunaliella tertiolecta* revealed no significant increase of lipids in various culture dilutions [50], suggesting species-specific differences in their glycerolipid metabolism.

Of particular note is that *M. neglectum* exhibited a comparatively high accumulation of biomass even within the nitrogen starvation period, performing clearly better than the model organism *C. reinhardtii* and the previously investigated *M. contortum *[19]. The neutral lipid productivity of *M. neglectum* determined in this work (52 mg l^-1^ day^-1^) is relatively high when compared to multiple other strains under photoautotrophic growth conditions [27], but appears low in comparison to other data considering *Nannochloropsis sp. *[15] and *Nannochloropsis gaditana *[21]. Lipid productivities of up to 204 mg l^-1^ day^-1^ were reported under nitrogen deprivation for *Nannochloropsis sp. *[15], while *N. gaditana* showed productivities of up to 310 mg l^-1^ day^-1^[21]. It is however important to note that a comparison of these numbers can be misleading, since the cultivation conditions applied in the *Nannochloropsis* work mentioned before were considerably different to the conditions applied here, with respect to varying initial biomass densities, differences in illumination and potential stress caused by outdoor cultivation conditions. This issue is further emphasized by the fact that another *Monoraphidium* strain, which was suggested as potential feedstock for biodiesel production before, showed lipid productivities of 149 mg l^-1^ d^-1^ in heterotrophic and only 7 mg l^-1^ d^-1^ in autotrophic conditions [24]. For accurate evaluation of *M. neglectum*, three control strains were used in this work, *C. reinhardtii*, *P. kessleri*, and *S. obliquus*. Higher TAG levels were reported for *S. obliquus* and *P. kessleri* strains in recent publications in comparison to our findings [17,51], indicating a clear potential to further optimize the lipid productivity of *M. neglectum* by systematic improvements of the culturing conditions, which was not in the focus of this work. As lipid productivities are highly dependent on biomass concentrations, higher productivities for *M. neglectum* are likely to be attainable through further optimization studies emphasizing optimised biomass densities and light penetration [17,28,51].

The lipid contents determined for the oleaginous control strains *P. kessleri* and *S. obliquus* in this work were in the same range as for *M. neglectum*, demonstrating that the potential of all three species for liquid biofuels production can be regarded as being similar. C18:1 and C16:0 were the major fatty acids that accumulated in *M. neglectum* after nutrient starvation, corresponding well to previous results from the related species *M. contortum *[19], suggesting similar mechanisms of lipid accumulation in both organisms. In particular, oleic acid is known to accumulate under nitrogen starvation in many species such as *Ankistrodesmus braunii*, *C. vulgaris, Chlorella zofingiensis*, and *S. obliquus *[17,52]. This is in contrast to *C. reinhardtii*, where more unsaturated fatty acids are common, contributing significantly to the more diverse fatty acid pattern under nitrogen starvation. This could indicate that, in *C. reinhardtii*, membrane recycling and conversion of polar lipids to triacylglycerols plays a more prominent role [45] when compared to *M. neglectum*.

The high levels of C18:1 and saturated C16 in *M. neglectum* under nitrogen starvation are a promising fatty acid profile for biofuel production which encourages the cultivation of this strain in large-scale concepts for the production of biofuels, preferably in outdoor bioreactors. This composition, however, would most likely require further blending with oil from other sources to meet the requirements for liquid biofuels such as biodiesel [1].

The productivity in outdoor cultivation conditions very much relies on the robustness of the strain used. Parameters defining robustness include tolerance to changing light intensity, temperature, pH, or salinity. Several studies have been performed on salt and pH tolerance of various microalgal strains [53-55]. Some adapted marine organisms such as *Dunaliella tertiolecta* tolerate salt concentrations up to three times higher than sea water and show an increased lipid content under these conditions [53]. These values were outstanding and could not be reached with other organisms, though some strains such as *Navicula sp.* are known to require at least salt concentrations similar to sea water to retain their highest biomass productivity [56]. In this context, *M. neglectum* exhibited robust phototrophic growth in salt concentrations up to 0.5% sodium chloride in the medium (Figure 2) which is comparatively high when compared to other freshwater strains [54]. This tolerance to salinity points towards efficient adaptation mechanisms to different salt concentrations in the investigated range. Together with the observed tolerance against high pH ranges (Figure 3), it is feasible to suggest that *M. neglectum* has robust growth characteristics likely suitable for outdoor cultivation.

A further distinct feature of *M. neglectum* is the increase in cell size when cultivated under brackish water conditions (Figure 2C) which could potentially reduce energy costs of harvesting during downstream processing [57].

Although currently no transformation strategy exists for this strain, the sequencing of its genome will allow the elucidation of regulatory regions which may become important in the construction of transformation vectors. The identification of the commonly used antibiotic hygromycin B as a selective agent within this work will also aid in future genetic manipulation attempts with this organism.

### The genome of *M. neglectum* reveals a highly diverse repertoire of genes encoding catalytic activities in carbohydrate and fatty acid metabolism

For accessing the full potential of this alga as a potential biotechnological production host, the genome of *M. neglectum* was sequenced, assembled and annotated and eventually compared to existing data derived from other green microalgae and oleaginous Heterokontophyta. It could be concluded that *M. neglectum* represents a highly interesting genome which can be used for comparative analyses to further elucidate parameters for efficient neutral lipid synthesis in microalgae. With an estimated genome size of 68 Mbp (Table 2), the *M. neglectum* genome is considerably larger than the genome of the Heterokontophyte *Nannochloropsis gaditana* (ca. 30 Mb, [21]), but smaller than the 121 Mb genome of the green alga *C. reinhardtii *[8]. However, *C. reinhardtii* (version 1.1) and *M. neglectum* have similar gene numbers, indicating that only a small fraction of the genome can be assigned to coding sequences. This percentage is 16.7% in *C. reinhardtii *[8] and slightly higher in the *M. neglectum* genome (25.1%)*.*

The genomic G+C content of 64.74% of *M. neglectum* is similar to that of *C. reinhardtii* and *Chlorella variabilis *[8,58], but considerably higher than that of *N. gaditana* (54% [21]). Lower G+C-contents have also been reported for *Cyanidioschyzon* and *Ostreococcus tauri* (see [8], and references therein).

The size of the chloroplast was determined to be 135,362 bp, therefore, within the typical range of chloroplast genome sizes of algae and plants [59]. The gene content of the chloroplast genome is similar to that of other known algal chloroplasts. Surprisingly, the mitochondrial genome differs significantly in length from other known algal genomes. With an estimated length about 96 kbp, at present the *M. neglectum* mitochondrial genome represents the largest known mitochondrial genome in algae. The largest stramenopile mitochondrial genome had been identified in the diatom *Phaeodactylum tricornutum* (77.4 kbp, [60]). Certain plant species were shown to harbour longer mitochondrial genomes, which can reach sizes of >2 Mbp, such as found in *Cucumis melo* and *Cucumis sativus *[37], considerably larger than *M. neglectum*. Moreover, the mitochondrial genome of *M. neglectum* also exhibits a rather small gene density, therefore, resulting in a similar or even lower gene number than observed in other algae, including *C. reinhardtii *[8] or *N. gaditana *[21].

Research using haploid-dominant algae has for decades benefitted from ease of transformation and rapid forward and reverse genetics with *C. reinhardtii* or *Volvox carteri* as prominent examples [61]. In contrast, the sequenced genome of *M. neglectum* was found to be diploid, similar to several other green algae like *Dunaliella salina* or *Haematococcus pluvialis* and several diatoms [61]. This finding indicates that, compared to *C. reinhardtii*, future genetic transformation strategies will need to address the emergence of hemizygous individuals. Alternatively, further elucidation of the life cycle of *M. neglectum* is recommended to allow potentially easier transformation if generation of haploid cells is possible.

The total number of predicted genes in the *M. neglectum* genome is 16,845, which is considerably higher than in *N. gaditana* (9,052 genes, [21]). A similar number of genes were predicted for *C. reinhardtii* (15,143 gene predictions before RNA sequencing [8]). It has to be noted that the determination of gene numbers is dependent on the prediction algorithm, its training sets, and the inclusion of other data sources such as transcriptome data. Transcriptome studies have not been performed on *M. neglectum* yet and are desirable to validate the predicted genes.

The differential analysis of the gene content of *M. neglectum, C. reinhardtii*, and *N. gaditana* via gene ontology (GO) allowed further insights into the degree of conservation and diversity of algal genomes. In all three investigated genomes, an over-representation of specific functional categories was found, including ATP binding, protein binding, and catalytic activity (Figure 7). These over-representations may indicate the trend to expand genomes towards regulatory mechanisms, which could be in particular the case for *C. reinhardtii*. Interestingly, *M. neglectum* exhibited a higher number of genes in categories related to fatty acid biosynthesis, lipid metabolic processes and carbohydrate metabolism, suggesting a higher versatility or functional redundancy when compared to the other two organisms. The reconstruction of the metabolic pathways, therefore, provided more detailed information.

### The reconstruction of lipid pathways in *M. neglectum* reveals new insights into fatty acid and neutral lipid synthesis

The reconstruction of the metabolic network concerning fatty acid and glycerolipid metabolism was a crucial precondition to interpret the biochemical data. This reconstruction was also valuable to gain important insights into the lipid metabolism of *M. neglectum* to identify potential genetic engineering targets in neutral lipid synthesis. The high number of homologous genes identified for acetyl CoA carboxylase (ACC), 3-oxoacyl-ACP reductase (KAR), and beta-ketoacyl-acyl-carrier-protein synthase (KAS1/2), suggests a more pronounced role of these factors for the lipid metabolism in *M. neglectum* compared to the green alga *C. reinhardtii*. This functional redundancy could promote the comparatively fast accumulation of fatty acids in *M. neglectum*, leading potentially to an intense use of the chloroplast pathway for producing TAG precursors.

Though the characterization of the *M. neglectum* genome also reveals the presence of a broad variety of desaturases, the fatty acid analysis of nitrogen starved cultures resulted in the identification of mainly palmitic acid (C16:0) and oleic acid (C18:1), contrasting to a high diversity of fatty acids in *C. reinhardtii*. The very distinct fatty acid production in *M. neglectum*, however, represents a very promising profile for biodiesel production from fatty acid methyl esters (FAMEs), where a high degree of saturation is desired [1]. Further analysis of the lipid metabolism identified the existence of a distinct gene coding for a palmitoyl-protein thioesterase and three further homologues for oleoyl-ACP hydrolases (Figure 8). Since thioesterases were reported to potentially reduce feedback inhibition in fatty acid biosynthesis in diatoms [62], the abundance of these enzymes could be considered as a potential bottleneck for efficient fatty acid production and consequently could be putative targets for improving the fatty acid production in microalgae by genetic engineering when genetic tools for *M. neglectum* become available.

To elucidate the role of the thioesterases in bypassing feedback inhibition, it will be of special interest to investigate their gene expression during nitrogen starvation. It has to be noted that the central carbon metabolism of diatoms shows some pronounced differences compared to green algae [63], therefore, the data from this work open up the opportunity for further comparative studies between Chlorophyta and diatoms to investigate the metabolic fluxes and their regulation towards fatty acid production, culminating in neutral lipid synthesis.

In addition to fatty acid synthesis, the abundance of triacylglcerols (TAGs) is a deciding factor for the suitability of a microalga for oil-based biofuel production.

The analysis of the *M. neglectum* genome and the reconstruction of the glycerolipid metabolism revealed the presence of only three DGAT type 1 and 2 homologs, which appears unusually low when compared to *C. reinhardtii *[44,45] or the oleaginous Heterokontophyte *N. gaditana *[21]. The comparably low number of DGAT homologs indicates that this pathway for TAG generation could *in vivo* be less adaptive in *M. neglectum*. Therefore, the DGAT genes represent highly interesting targets for comparative functional studies. Together with the distinct accumulation of C16:0 and C18:1 fatty acids in *M. neglectum* under nitrogen starvation, this finding furthermore points towards the important role of the chloroplast pathway for the synthesis of neutral lipid precursors [64].

### *M. neglectum* can serve as a model organism to elucidate the distinct role of PDAT enzymes in TAG accumulation and homeostasis

When the acyl-Co independent mechanism of TAG generation is addressed, the presence of two PDAT homologues was confirmed with one of the candidates (Mneg_03131.t1) carrying a distinct functional acyltransferase domain. Interestingly, this is, to our knowledge, the first time that such a PDAT has been identified in the genome of an oleaginous green microalga. While PDAT homologues could be found in the transcriptome of the marine strain *Dunaliella tertiolecta*, they appear to be absent in *Neochloris oleoabundans *[43].

It is of particular note that the lipid accumulation pattern of *M. neglectum* exhibits several similarities to the oleaginous green microalga *N. oleoabundans* such as the increase of FAMEs and specifically of C18:1 (up to about 55%), while retaining a similar percentage of C16:0 (approx. 23%) of the fatty acid composition under nitrogen starvation [43]. Since PDAT is potentially not present or expressed in *N. oleoabundans*, a comparison of these two closely related species represents a highly interesting target for further investigations to elucidate its metabolic function at normal growth conditions and under different stresses. These findings also shed light on the diversity of lipid metabolism in the branch of green algae, as it has been noted before [11].

## Conclusions

This investigation provides a foundational analysis of *M. neglectum*, a member of the Selenastraceae, which exhibits robust growth characteristics as well as relatively high lipid yields. Biomass and lipid production analyses presented in this work clearly demonstrate that *M. neglectum* has a high potential to serve as a production strain for future liquid biofuel concepts. This strain demonstrates favourable characteristics for cultivation including robust photoautotrophic growth, high neutral lipid contents containing an interesting profile of fatty acids during nitrogen starvation, pH tolerance, as well as a large cell phenotype at higher salt concentrations. A further increase of lipid yields can be expected when growth conditions are systematically optimised.

Genomic annotation and feature analysis elucidated key features of the lipid synthesis pathway, providing new insights into the evolution and physiological differences regarding the conversion of carbon dioxide into energy-dense glycerolipids in microalgae. The nuclear genome exhibits comparatively high redundancy in fatty acid synthesis processes, while the number of homologues involved in the acyl-CoA dependent TAG generation is considerably smaller, therefore, presenting potential targets for future genetic engineering approaches. The genomic analyses presented in this work will aid in the identification of potential bottlenecks for neutral lipid synthesis and provide the basis for future genetic engineering strategies.

## Methods

### Cultivation conditions

*Monoraphidium neglectum* (SAG 48.87) and *Scenedesmus obliquus* (SAG 276-6) were obtained from the Algae Collection in Göttingen, Germany, while *Chlamydomonas reinhardtii* (CC1690) was obtained from the *Chlamydomonas* Resource Center, University of Minnesota, US, and *Parachlorella kessleri* (211/11H) from the CCAP, United Kingdom. Cultivations were performed in Provasoli based minimal media (ProF) as described previously [19]. Cells were cultivated in 20 ml minimal media in Erlenmeyer flasks on a rotary shaker and used for inoculation of 3 l roller flasks adjusted to an OD_750_ of 0.05. Growth was monitored by daily OD_750_ measurements as well as biomass development and manually counted cell number. Cultivations were performed under constant illumination with white light at intensities ranging between 350-400 μmol photons m^-2^ s^-1^. Media were aerated with air enriched with 3% CO_2_. After 3 days, cells were harvested and transferred to 400 ml batch cultures.

### Nitrogen starvation and stress tolerance

For the induction of lipid accumulation, cultures were transferred to media with the same composition but lacking a nitrogen source as described previously [19]. To assess general cultivation conditions of the second stage a further batch of 1:4 diluted cultures was transferred to nutrient replete conditions as control. Biomass was harvested after five days of cultivation, lyophilized and stored at -80°C prior to further investigations.

Salt tolerance was investigated in liquid cultures containing 0.1, 0.5, 1.0 and 2.0% (w/v) sodium chloride. Performance of cultures was monitored by measurements of OD_750_ and the dry biomass weight. Before cultures reached the stationary phase, the biomass was harvested, lyophilized and subjected to lipid extraction and chromatographic analysis.

The pH sensitivity of *M. neglectum* was tested in plate assays. 5 ml of cell suspension (adjusted to OD_750_ =0.2) were spotted on agar plates containing Provasoli freshwater medium with additional 0.59 μM thiamine, 4.1 nM biotin and 0.6 nM cobalamin. For pH 3.0, the Provasoli medium (adjusted to pH 3.0) and agar were separately autoclaved and combined after cooling down to about 60°C, while for pH 5.0 - 7.5 Provasoli media were adjusted to the respective pH before agar was added and autoclaved. For plates with pH 10, each stock solution for Provasoli (with CaCl_2_ diluted to 1/10 of the original concentration) as well as double distilled water containing the agar and sodium chloride were autoclaved separately and combined at a temperature of about 60 - 70°C. For mixotrophic cultivation on agar, 10 g L^-1^ glucose was added to media after autoclaving by filter sterilisation. Vitamins were added after media were cooled down to about 60°C.

### Lipid extractions and chromatographies

Extractions were performed in two technical replicates per biological replicate from 50 mg lyophilized biomass. After homogenization (3 x 30 s at 6,500 rpm using a Precellys 24, Peqlab, Erlangen, Germany), the total lipids were extracted according to a modified Folch protocol [65] using a total of 4 ml methanol and 8 ml chloroform. Contaminants were removed by washing the extract with 3 ml of deionised water. From the dried total lipid extract, column chromatographies were performed to separate the neutral from the polar lipid fraction as described elsewhere [66].

### FAME analysis

Neutral and polar lipids were transesterified to fatty acid methyl esters (FAME) as described previously [19]. Spectra analysis was performed with Xcalibur. The peak identity was confirmed by comparison of retention times and mass spectra to a 37 FAME mix (47885-U, Supelco). Unidentified peaks were analysed on their mass spectra and attributed to fatty acids according to their fragmentation pattern. To determine the relative amount of fatty acids within the total lipid fraction, 50 μg C17-triacylglycerol (glyceryl triheptadecanoate, Sigma-Aldrich, Steinheim, Germany) were added to each sample as an internal standard. Total ion chromatograms were recorded and used to calculate the relative abundances of the individual fatty acid after normalization to the internal standard (set to 100, dry biomass basis).

### DNA isolation and sequencing

DNA was extracted using the cetyltrimethylammonium bromide method as reported previously [67]. After an RNAse digest (Ribonuclease A, Roth, Germany), the quality was controlled in a 1% agarose gel.

The sequencing was performed on an Illumina MiSeq machine with sequencing libraries prepared using the Illumina Nextera® DNA Sample kit. DNA fragments of a size between 500 and 700 base pairs were cut from an agarose gel and purified with a MinElute Gel Extraction Kit (Qiagen). DNA amount and quality were monitored on an Agilent Bioanalyzer. The sequencing was performed using the MiSeq Reagent Kit v2 (Illumina) with 2 × 250 cycles.

### Genome assembly and gene annotation

All reads obtained by genome sequencing were assembled to contigs and scaffolds using the Newbler assembler version 2.6 (Roche) with settings for heterozygous genomes. Nuclear and organelle genomes were assembled manually using the compatible finishing package Consed version 23.0 [68].

The annotation of the three genomes (chloroplast, mitochondrion and nuclear) was done by a specific annotation pipeline, which consists of three steps. All potential genes were predicted by two *ab initio* gene prediction tools: Augustus [31] with the *Chlamydomonas reinhardtii* genome as training set and in parallel with GeneMark-ES (version 2) [32], which combines GeneMark.hmm for prediction of eukaryotic genomes with a self-training procedure. In addition, a protein alignment with all *C. reinhardtii* proteins was performed. To evaluate over 34,000 predicted genes the software EVidenceModeler (EVM) [33] was used to filter the gene set and to eliminate putative false positive predictions. For that purpose we assigned different weightings for the different prediction outputs. Augustus and GeneMark were assigned with the same higher weight (2) and the protein alignment a lower weight (1). Ribosomal RNAs were identified by using the RNAmmer 1.2 server [69] and tRNAs were determined by tRNAscan-SE version 1.21 [70,71]. Organellar genomes were analysed and printed with OGDRAW [30]. All gene information were integrated to the annotation platform GenDBE, which is a modification of GenDB for the annotation of eukaryotic genomes [34]. GenDBE allows the manual curation of gene specific annotation in addition to the visualisation of gene order.

This Whole Genome Shotgun project has been deposited at DDBJ/EMBL/GenBank under the accession AYTC00000000. The version described in this paper is version AYTC01000000.

For genome comparison based on GO categories gene specific GO-terms were obtained by analysing all genes of the three genomes *C. reinhardtii*, *N. gaditana* and *M. neglectum* in Interpro [41].

### Phylogenetic analysis

The evolutionary history was inferred by using the Maximum Likelihood method based on the Tamura-Nei model [72], with the tree of the highest log likelihood (-6385.4692) shown. Initial trees were obtained by the Neighbor-Joining method to a matrix of pairwise distances, estimated using the Maximum Composite Likelihood (MCL) approach. The tree is drawn to scale. The branch lengths are measured in number of substitutions per site. Numbers indicate bootstrap values after 5,000 replications. The analysis involved 12 nucleotide sequences with a total of 1284 positions in the final dataset. Evolutionary analyses were conducted in MEGA5 [73].

### Metabolic pathway reconstruction

Pathway reconstruction was performed for FA and TAG synthesis by means of PathwayTools and PRIAM. The KEGG pathway proposed on the gene set of *M. neglectum* by GenDBE was analysed using PRIAM (database profile of March 2013) [34,74]. Therefore, the E.C. numbers of the proposed KEGG pathway were used as basis for the PRIAM search to identify corresponding genes with an E-value cutoff of 1*10^-10^.

## Abbreviations

ACP: Acyl carrier protein; BLAST: Basic local alignment search tool; CoA: Coenzyme A; DAG: Diacylglycerol; DNA: Deoxyribonucleic acid; EVM: EVidence modeler; FA: Fatty acid; NaCl: Sodium chloride; TAG: Triacylglycerol; TRIS: Tris(hydroxymethyl)aminomethane.

## Competing interests

The authors declare that they have no competing interests.

## Authors’ contributions

The research was designed by CB, AAD, JHM, JK and OK. CB, AAD, JW, MG, KL, OBK performed the research, with the main part of this work carried out by the first author, CB. CB performed growth analysis, lipid analysis and FAME profiling under nutrient replete conditions and nitrogen starvation, was involved in genome sequencing, performed phylogenetic analysis, and *in silico* reconstruction of lipid metabolism, prepared therefore figures and tables and drafted the manuscript. JW investigated growth under nitrogen starvation and performed lipid analysis. MG performed investigations of salt tolerance, KJL of resistance to antibiotics and OBK of pH tolerance. Genome sequencing was performed by AA, genome assembly and annotation by AA and AAD. The GENDBE project was created and maintained by OR and AG. Manual gene assignments and comparison of genomes was carried out by AAD. AAD prepared figures and tables related to genome assembly and metabolic reconstruction. Data analysis and interpretation: CB, AAD, JHM, OK. Manuscript writing: CB, AAD, JK, JHM, OK. All authors read and approved the final manuscript.

## Supplementary Material

Additional file 1: Figure S1 Neutral lipid productivity of *M. neglectum* when compared to *C. reinhardtii* for the overall production period: 3 days growth under nutrient replete conditions (OD_750_ = 0.05 - 0.06 for inoculation), 5 days starvation under nitrogen deficiency (+N, preculture and –N, high OD). Click here for file

Additional file 2: Figure S2 Fatty acid abundances of *M. neglectum* and *C. reinhardtii* grown under nutrient replete (+N) and nitrogen starvation (-N) conditions as determined via GC-MS. Error bars show standard deviation (n = 4). Click here for file

Additional file 3: Figure S3 Read versus contig plot reveals diploid character of the *M. neglectum* genome. Click here for file

Additional file 4: Figure S4 Comparison of chloroplast genomes of *C. reinhardtii*, *M. neglectum* and *N. gaditana*. Click here for file
