# Supplementary figures and images for: Reconstruction of the lipid metabolism for the microalga Monoraphidium neglectum from its genome sequence reveals characteristics suitable for biofuel production
Source: BMC Genomics. 2013 Dec 28;14:926. doi: 10.1186/1471-2164-14-926 (PMC3890519; doi:10.1186/1471-2164-14-926)

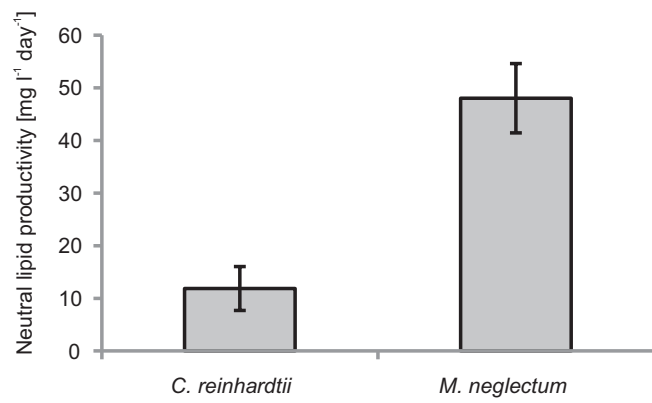

Supplement: Additional file 1: Figure S1 — Neutral lipid productivity of M. neglectum when compared to C. reinhardtii for the overall production period: 3 days growth under nutrient replete conditions (OD750 = 0.05 - 0.06 for inoculation), 5 days starvation under nitrogen deficiency (+N, preculture and –N, high OD). [file 1471-2164-14-926-S1.pdf]

*M. neglectum*

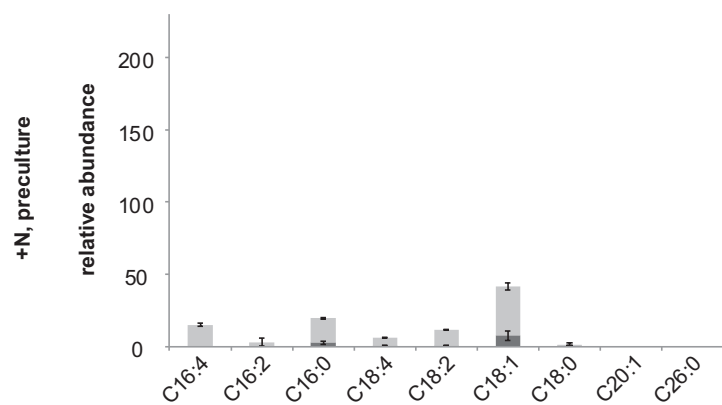

*C. reinhardtii*

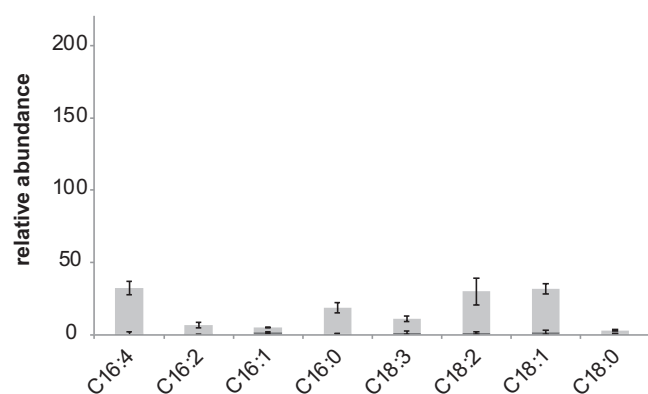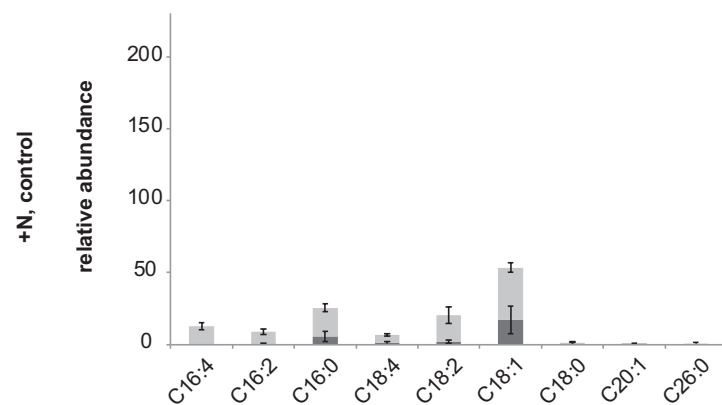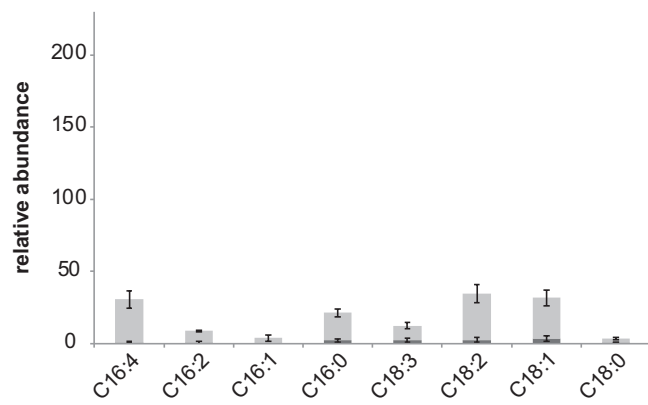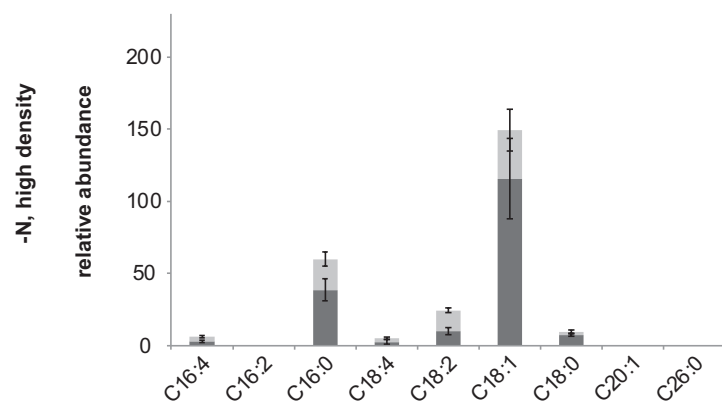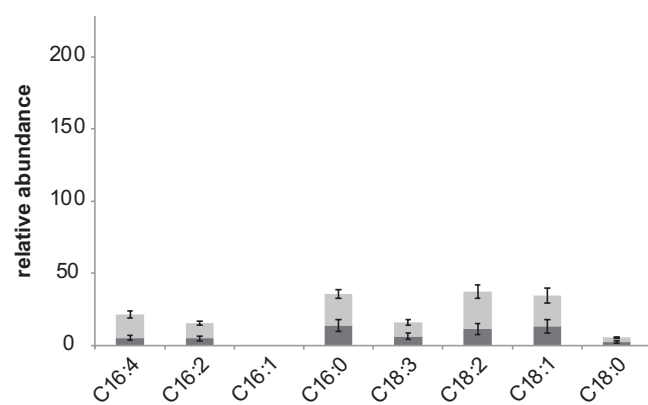

■ polar lipids

■ neutral lipids

Supplement: Additional file 2: Figure S2 — Fatty acid abundances of M. neglectum and C. reinhardtii grown under nutrient replete (+N) and nitrogen starvation (-N) conditions as determined via GC-MS. Error bars show standard deviation (n = 4). [file 1471-2164-14-926-S2.pdf]

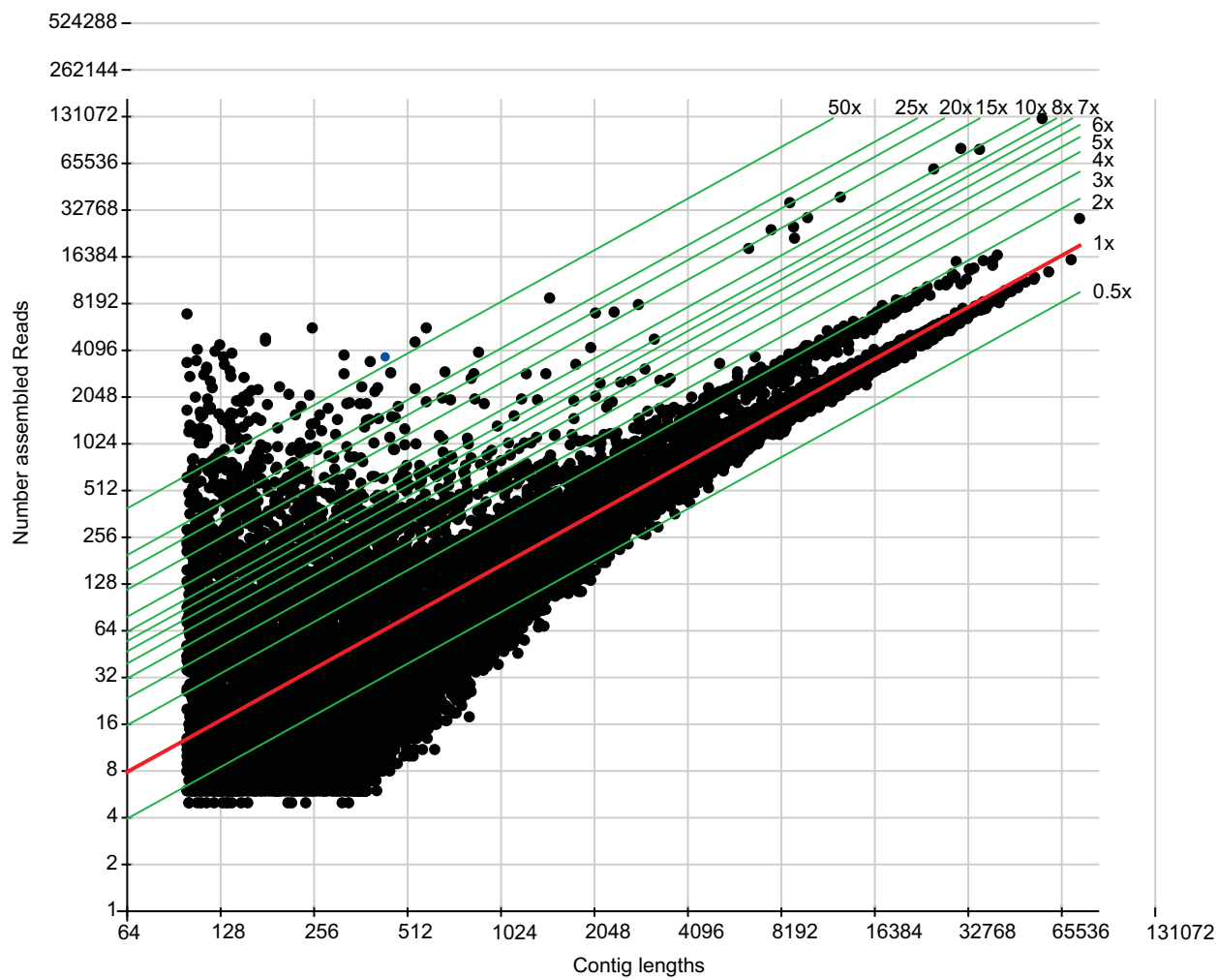

Supplement: Additional file 3: Figure S3 — Read versus contig plot reveals diploid character of the M. neglectum genome. [file 1471-2164-14-926-S3.pdf]
