# Supplementary material for: Reconstruction of the lipid metabolism for the microalga Monoraphidium neglectum from its genome sequence reveals characteristics suitable for biofuel production
Source: BMC Genomics. 2013 Dec 28;14:926. doi: 10.1186/1471-2164-14-926 (PMC3890519; doi:10.1186/1471-2164-14-926)
Supplement: Additional file 4: Figure S4 — Comparison of chloroplast genomes of C. reinhardtii, M. neglectum and N. gaditana. [file 1471-2164-14-926-S4.pdf]

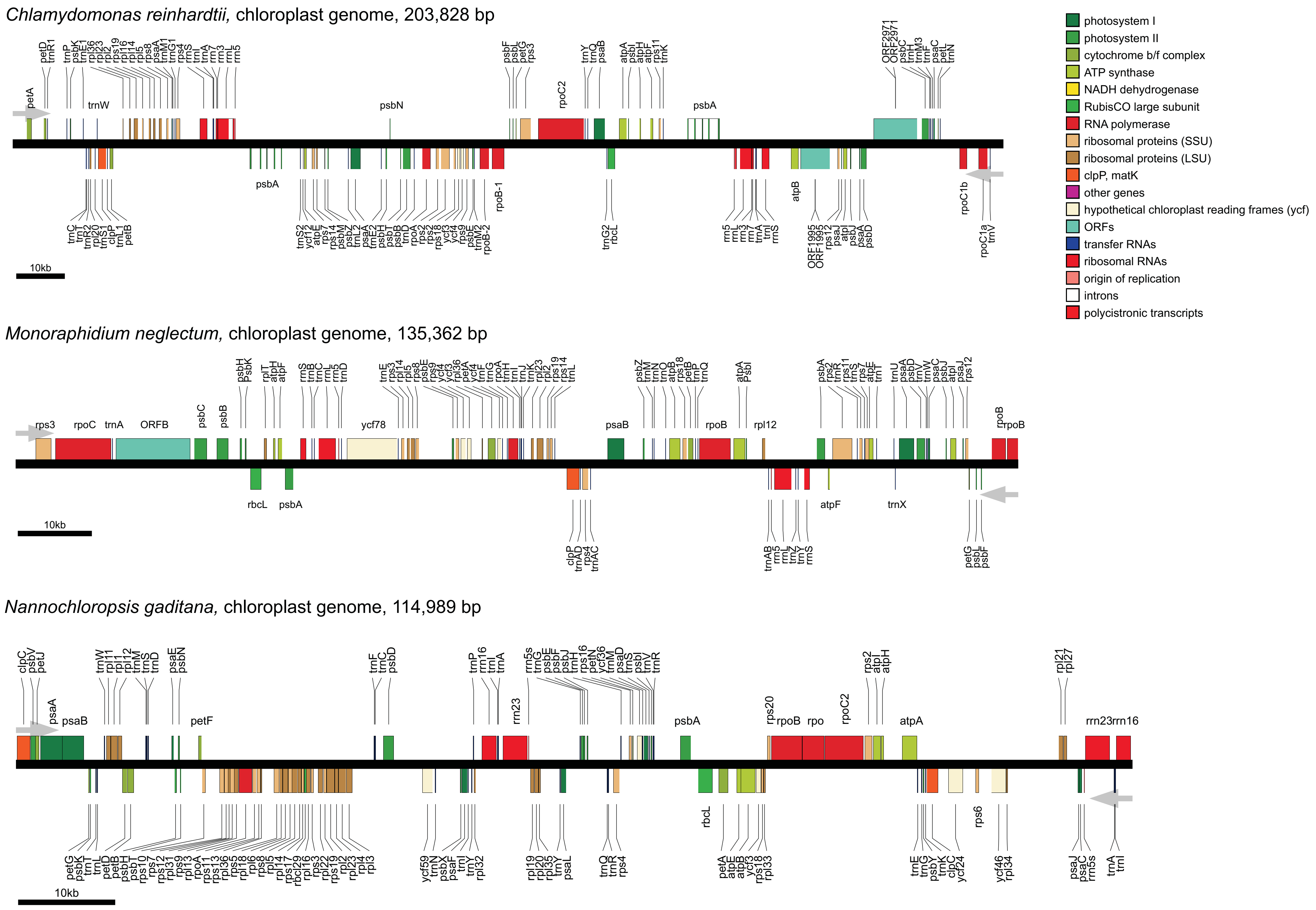

Chlamydomonas reinhardtii, chloroplast genome, 203,828 bp
photosystem I
photosystem II
cytochrome b/f complex
ATP synthase
NADH dehydrogenase
RubisCO large subunit
RNA polymerase
ribosomal proteins (SSU)
ribosomal proteins (LSU)
clpP, matK
other genes
hypothetical chloroplast reading frames (ycf)
ORFs
transfer RNAs
ribosomal RNAs
origin of replication
introns
polycistronic transcripts
trnW
psbN
psbA
psbA
10kb
Monoraphidium neglectum, chloroplast genome, 135,362 bp
rps3
rpoC
trnA
ORFB
ycf78
psaB
rpoB
rpl12
rbcL
psbA
atpF
trnX
10kb
Nannochloropsis gaditana, chloroplast genome, 114,989 bp
psaB
petF
psbA
rpoB
rpo
atpA
rrn23rrn16
10kb
